# Neurological Soft Signs in Schizophrenia: An Update on the State- versus Trait-Perspective

**DOI:** 10.3389/fpsyt.2017.00272

**Published:** 2018-01-08

**Authors:** Silke Bachmann, Johannes Schröder

**Affiliations:** ^1^Department of Psychiatry, Psychotherapy, and Psychosomatics, University Hospitals of Halle (Saale), Halle, Germany; ^2^Clienia Littenheid AG, Hospital for Psychiatry and Psychotherapy, Littenheid, Switzerland; ^3^Section of Geriatric Psychiatry, Department of General Psychiatry, Center for Psychosocial Medicine, University of Heidelberg, Heidelberg, Germany

**Keywords:** schizophrenia, neurological soft signs, longitudinal, follow-up, state, trait, review

## Abstract

**Background:**

Neurological soft signs (NSS) represent minor neurological signs, which indicate non-specific cerebral dysfunction. In schizophrenia, their presence has been documented extensively across all stages of the disease. Until recently, NSS were considered an endophenotype or a trait phenomenon. During the past years, however, researchers report fluctuations of the NSS scores.

**Aims:**

To further clarify the question whether NSS exhibit state or trait components or both, studies that have investigated NSS longitudinally were reviewed.

**Method:**

Studies which have assessed NSS longitudinally in adults suffering from schizophrenia, were searched for. The time frame was January 1966 to June 2017. Studies on teenagers were excluded because of interferences between brain maturation and pathology.

**Results:**

Twenty-nine follow-up studies were identified. They included patients during different stages of their illness and mainly used established instruments for NSS assessment. Patients with a first episode or a remitting course predominantly show a decrease of NSS over time, whereas a worsening of NSS can be found in the chronically ill. It was shown that change of NSS total scores over time is predominantly caused by motor system subscales and to a lesser extent by sensory integration scales. With respect to medication, the majority of studies agree on a relationship between medication response and improvement of NSS while the type of antipsychotic does not seem to play a major role. Moreover, where information on side-effects is given, it does not favor a strong relationship with NSS. However, NSS seem to correlate with negative and cognitive symptoms.

**Conclusion:**

Studies manifest a conformity regarding the presence of NSS in schizophrenia patients on the one hand. On the other hand, fluctuations of NSS scores have been widely described in subgroups. Taken together results strongly support a state-trait dichotomy of NSS. Thus, the usage of NSS as an endophenotype has to be called into question.

## Introduction

It is generally accepted that neurological soft signs (NSS) are present in schizophrenia patients. These neurological abnormalities do not reflect hard pathology, i.e., localized, nuclear, or primary tract lesions (1–3) and are, therefore, labeled “soft.” Overall, in schizophrenia patients, distinctive NSS consistently pertain to motor coordination, motor sequencing, and sensory integration [review by Boks et al. (4)], but also to eye movements and developmental reflexes (3, 5). In first-episode (FE) patients NSS mostly strike motor coordination, motor sequencing, and developmental reflexes [review by Dazzan and Murray (6)].

Yet, the presence of NSS is not specific to schizophrenia. In early studies, they were detected in 5% (7, 8) to 50% (9, 10) of healthy individuals, depending on the instrument used. NSS can be found in patients with mood disorders (11), obsessive–compulsive disorder (12), and personality disorders (13). However, the incidence of NSS is higher in schizophrenia compared to other psychiatric disorders [review by Dazzan and Murray (6)].

Also, compared to healthy controls (HC) NSS occurrence in schizophrenia patients is more pronounced with regard to both quantity and quality (14). Several reviews have summarized the respective research results on schizophrenia patients (4, 6, 15–18). These reviews also emphasize that NSS and abnormal movements similar to tardive dyskinesia are not sequelae of neuroleptic medication but an integral part of the disease as has been described by Kraepelin for the first time in the end of the nineteenth century (19).

Predominantly, NSS have been looked at as endophenotypes, i.e., hardcopies of the genetic liability and thus as a trait phenomenon. In fact, much support for this notion derives from the literature on neuroleptic-naïve patients. The review by Wolff and O’Driscoll (20) reports a rate of approximately 25–33% NSS in this group and elevated rates in high-risk subjects with an emphasis on motor abnormalities.

Another strong piece of evidence supporting the trait aspect of NSS stems from FE studies [review by Dazzan and Murray (6)], where the reported prevalence ranges from 20 to 97% of patients. In contrast, NSS can be ascertained in up to 60% of patients treated with antipsychotic medication compared to less than 1% in healthy subjects (14). Yet another argument underpinning the trait hypothesis is provided by the literature on increased NSS in first-degree relatives (20) and more specifically by twin studies (21–24) as well as by studies on patients’ off-spring, i.e., high-risk individuals (25–27). Interestingly, NSS abnormalities are comparable in patients and their relatives, they affect motor coordination, motor sequencing, and sensory integration.

On the whole, the literature thus offers good evidence in favor of NSS representing a trait phenomenon which again is in line with the neurodevelopmental hypothesis (28).

Despite the aforementioned evidence, a number of studies present arguments in favor of a state feature being present in NSS. First and foremost, follow-up studies report clearly marked fluctuations regarding the expression of NSS during the disease course (29–31). These fluctuations concern quality more than quantity (number) of abnormal NSS. Thus, single NSS may reach a higher level during acute phases of the disease and return to a baseline thereafter. However, this baseline does not compare to healthy individuals but rather to healthy first-degree relatives (22).

The documentation of a NSS state component may bear the following impact:

1. it may argue in favor of the existence of neuronal regeneration processes in individuals suffering from schizophrenia.
2. it may represent an argument for the necessity to supplement the neurodevelopmental hypothesis.
3. it may—together with fluctuations in symptomatology—hint at a third underlying factor influencing the disease process.

This paper aims to raise the question whether NSS are constituted by trait properties only or whether there is another property, namely state component, or whether NSS are even Janus-faced. This state-trait discussion can only be led with reference to follow-up studies.

Moreover, this paper represents an update on our meta-analysis (32), where only studies were incorporated which gave sufficient statistical data, so that several meaningful longitudinal studies were not included.

## Methods

We performed a literature search of the period between January 1966 and June 2017 in the following databases: PsycNET, PSycINFO, Psyndex, and SCOPUS which includes PubMed. We used the following terms: schizophren* AND neurology* OR soft sign OR motor AND follow-up OR longitudinal OR Verlauf (German word for course, used in the German database Psyndex only). In addition, we examined references from all articles identified. Moreover, we excluded literature on children and adolescents because NSS are present at birth and decrease over time at least until puberty. They are, thus, correlated with brain maturation (33), reaching a “normal” level only in early adulthood.

## Discussion

### Patients, Instruments, Follow-up Period

We identified 26 studies (see Table 1) which had performed follow-up assessments of patients diagnosed with schizophrenia or related disorders. These studies were presented by 18 groups, 3 of which (29, 31, 37–39, 41–43, 47, 52, 53) had carried out two to five studies each. Two other groups published two to three papers each on different follow-up examinations of one patient samples (30, 48–50, 58). Therefore, the latter data were merged for this review’s purpose.

**Table 1:** Longitudinal studies on patients with schizophrenia in alphabetical order.

Reference	Subjects gender handedness controls	Age	Follow-up (weeks)	Instruments	First NSS assessment	Medication	Clinical course	Side-effects (SE)	NSS
Bachmann et al. (31)	39 first-episode (FE) 18 m, 21 f	27 (7.7)	60 (6.4)	HD 2 raters IRR 0.88	Following clinical stabilization	Atypicals	PANSS t0: 52.4 (25.6) t1: 52.0 (12.4)	AIMS Barnes SARS	t0: 15.7 (7.1) t1: 10.1 (7.9) *p* < 0.001
Germany	handedness: 20 r, 19 mixed			PANSS DSM-IV			21 R 18 NR	no SE	R t0: 17.3 (6.8) t1: 7.2 (5.8)
	22 healthy controls (HC)	28 (3.8)	40 (5.6)						NR t0: 13.8 (7.2) t1: 13.5 (8.7)

Beher (34)	17 FE 9 m, 8 f	32.7 (7.2)	321 (104)	NES modified 1 rater	Antipsychotic naïve	“Antipsychotics”	Positive 24.9 (5.5) 14.1 (9.4) *p* < 0.001	AIMS SARS	Motor coordination t0: 2.0 (2.1) t1: 1.9 (2.1)
				PANSS DSM-IV			Negative 18.3 (8.6) 18.0 (7.7)	no SE	Sequential movement t0: 4.4 (3.1) t1: 4.1 (2.8)
							General 38.5 (8.8) 31.6 (8.7*)* *p* < 0.05		Sensory integration t0: 4.3 (3.4) t1: 2.3 (2.2)
India									Primitive reflexes t0: 2.8 (2.4) t1: 1.6 (1.5)

Boks et al. (35)	29 FE	26.9 (6.3)	104 (^a^)	NES 2 raters ICC 0.66	“After first episode”	15 atypicals 11 typicals 3 none	t0: 57.0 (17.4) t1: 52.9 (15.6)	n.d.	t0: 7.5 (7.1) t1: 8.9 (5.5)
The Netherlands	18 m, 11 f handedness^a^			PANSS DSM-IV			3 groups: Increased Unchanged Decreased		n.s.

Buchanan et al. (36)	31 CH 21 m, 10 f	34.1 (6.8) 34.6 (9.1)	10 (^a^) 10 (^a^)	NES 2 raters IRR 0.81–0.98	After 6 weeks fluphenazine, prior to double blind phase	16 clozapine 15 haloperidole (double blind)	t0: 11.4 (5.8) t1: 9.4 (5.2)	SARS TD	t0: 16.2 (5.9) t1: 16.8 (8.2)
USA				BPRS DSM-III-R			t0: 12.6 (5.3) t1: 12.0 (5.1)		t0: 14.5 (6.3) t1: 15.1 (7.5)

Chan et al. (37)	109 FE 69 m, 78 f at t0	Neg. sym. 22.3 (4.1)	26 (^a^)	CNI 3 raters ICC 0.85–0.91	^a^	About 90% atypicals	Negative symptoms 60.9 (13.9)	AIMS Barnes SARS	Negative symptoms 7.1 (3.2)
									No negative symptoms 5.6 (3.1)
	Handedness: 137 r, 8 l at t0	No neg. sym. 21.7 (3.8)	52 (^a^)	PANSS DSM-IV Logical Memory			No negative symptoms 42.2 (14.9)	Unchanged	HC 2.8 (2.2)
China	62 HC 32 m, 30 f 60 r, 2 l	21.2 (1.9)		Visual Reproduction Letter-Number Span Test Verbal fluency WCST					Motor Coordination 3.68 (1.98) 2.64 (1.88) <0.001 1.34 (1.28)
									Sensory Integration 2.26 (1.50) 1.99 (1.40) <0.001 0.74 (1.13)
									Disinhibition 1.19 (0.97) 0.98 (0.93) *p* < 0.05 0.68 (0.65)

Chen et al. (38)	43 CH 30 m, 13 f	48.9 (8.9)	152 (^a^)	CNI 2 raters ICC 0.45–0.95	Chronic	Typicals	t1: 27.6 (6.7) t2: 28.3 (5.9)	^a^	Motor Coordination t0: 17.5 (21.1) t1: 26.0 (24.8)
China	40 HC	48.4 (8.4)		BPRS DSM-III-R					Sensory Integration t0: 14.4 (16.5) t1: 25.4 (22.7)
									Disinhibition t0: 11.4 (11.8) t1: 24.5 (19.0)

Chen et al. (39)	93 FE 42 m, 51 f	31.2 (9.6)	6 (^a^) 52 (^a^) 104 (^a^) 156 (^a^)	CNI 2 raters ICC 0.93	Acute phase	48 antipsychotic naïve 45 max. 7 d low dose haloperidol	^a^	AIMS Barnes SARS	t0: 1.87 (2) t4: 1.45 (2.2)
China	68 HC			PANSS DSM-IV				Unchanged	

Cuesta et al. (40)	77 FE 53 m, 24 f	30.1 (10) 30.6 (6.2)	t1: 4(^a^) t2: 26 (^a^)	NES 2 raters ICC 0.71–0.99	Acute Antipsychotic naïve	Risperidone Olanzapine Both None	SAPS t0: 10.1 (3.5) t2: 1.6 (2.1)	^a^ 31% clinically meaningful change	t0: 17.1 (9.4) t1: 12.0 (7.9) t2: 9.9 (6.8)
				SAPS			SANS		
Spain	28 HC 19 m, 9 f			SANS DSM-IV			t0: 8.0(5.9) t2: 4.7(4.9)		

Emsley et al. (41)	66 FE m 31, f 35	28.1 (8.5)	t1: 13(^a^) t2: 26 (^a^) t3: 52 (^a^)	NES: 13 items 1 rater	60 antipsychotic naïve	“very low doses of haloperidol”	t0: 92.1 (16.2)t1-t3:^a^	AIMS^a^ Barnes^a^ SARS^a^	t0: 6.7 (3.7) t1-t3:^a^
South Africa				PANSS DSM-IV Cognition	6 max. of 4 weeks of antipsychotic medication				“no significant change over time”

Emsley et al. (42)	126 FE 93 m, 33 f	24.1 (6.6)	t1: 26 (^a^) t2: 52 (^a^)	NES: 13 items 3 raters IRR >0.9	Max. 4 weeks of antipsychotic medication	Flupenthixol p.o. followed by i.m.	94.8 (16.5) 52.9 (17.9) *p* < 0.0001	ESRS^a^	15.02 (7.99) 10.72 (6.71) *p* < 0.0001
South Africa				PANSS DSM-IV MATRICS MCCB					

Jahn et al. (43)	82 (18–50) CH 28 R 26 FE 22, unclear 6	29.6 (7.0)	t1: 2(^a^)	BMS 1 rater	Subacute phase of illness	42 typicals 33 CLZ 3 CLZ and typical 3 none	39.0 (24–87)	AIMS Barnes SARS	5.50 (^a^)
Germany	m 55, f 27			BPRS DSM-III-R WCST modified				Correlation of SARS and motor signs	Improving or stable patients: significant decrease in motor signs

Madsen et al. (44)	18 FE 11 m, 7 f	28.5 (20–41)	260 (5 years)	Extended standard neurological examination	At first admission	13 “antipsychotics”	SANS t0: 6.0 (0–11) t1: 3.0 (0–9)	Hans rating scale^a^	Deterioration in male patients, in patients with OC, with genetic load, without remission
Denmark	10 HC 5 m, 5 f	28 (23–38)	1 rater PSE-9 SANS, SAPS ICD-10		5 none	SAPS t0: 6.5 (2–14) t1: 5.5 (1–22)		

Mangot and Sawant (45) India	40 FE 21 m, 19 f	35.5 (11.9)	24 (^a^) 52 (^a^)	NES 1 rater PANSS ICD-10	At first admission Antipsychotic naïve	Risperidone	^a^		t0: 8.5 (7.1) t1: 5.5 (5.8) *p* < 0.001 t2: 3.3 (4.1)

Mittal et al. (46)	19 CH 19 m	36.3 (5.4)	6 (^a^)	Quitkin scale 1 rater	Min. 6 weeks off medication	Haloperidol	t0: 34.3 (2.1) t1: 22.4 (2.2)	^a^	t0: 6.3 (0.9) t1: 5.3 (0.8)
USA				BPRS DSM-III-R	min. BPRS score 30		*p* < 0.01		*p* < 0.05

Ojagbemi et al. (47) Nigeria	66 FE 36 m, 30 f	28.7 (6.4)	t1: 26 (^a^) t2: 52 (^a^)	NES 1 rater PANSS DSM-IV	Antipsychotic naïve except for 5 patient	Flupenthixol dec./i.m.	^a^	ESRS	t0: 21.5 (11.1) t1^a^

Prikryl et al (48–50)	92 FE all m	23.1 (5.7)	^a^ 52 (^a^)	NES 1 rater	Acute	Atypicals in 69%	All t0: 97.4 (22.0) t1: 58.4 (15.3) t2: 51.5 (19.0)	^a^	All t2: 6.79 (6.58)
	68 of this sample at 4 years follow-up		208 (^a^)	PANSS ICD-10			72 R t0: 97.6 (22.5) t1: 57.9 (14.4) t2: 43.8 (11.1)	72 R t0: 5.3 (5.9) t1: 2.7 (3.4); p < 0.01 t2: 4.4 (4.5)
								20 NR t0: 6.5 (4.1) t1: 4.2 (4.1); *p* < 0.05 t2: 10.1 (7.6)
Czech Republik							20 NR t0: 88.4 (19.9) t1: 53.0 (14.9) t2: 84.7 (21.6)		t0/t1/t2:non-rem > rem; *p* < 0.05/ < 0.005

Scheffer (51)	18 FE 21 m, 8 f	22.8 (4.2)	6 (^a^)	NES 2 raters ICC >0.80	Acute	Typicals	^a^	ESRS^a^	t0: 12.1 (5.3) t1: 13.2 (6.6)
					16 antipsychotic naïve	Clinical needs			
USA	50 HC	21.7 (4.0)		BPRS DSM-III-R					

Schröder et al. (29)	50 SCZ: 27 CH 23 R m:f^a^	36 (12.1) 28.9 (8.9)	t1: remission (variable)	HD 2 raters IRR 0.85	Acute	17 drug-naïve	^a^	SARS	t0: CH 27.8 (9.2) R 23.5 (8.3)
						clinical needs: 29 typicals 21 clozapine		Uncorrelated	
Germany	34 HC	25.7 (3.18)		BPRS DSM-III					t1: CH 22.1 (7.1) R 13.0 (4.7)

Schröder et al. (52)	32 CH and R	32 (9)	Variable	HD 2 raters IRR 0.85	Acute	Clinical needs	t0: 46.1 (7.3) t1: 32.1 (5.7)	^a^	t0: 21.3 (8.3) t1: 11.5 (5.7)
	^a^HC	27 (2)							*p* < 0.005
Germany				BPRS DSM-III SKT Tower of Toronto WCST					

Schröder et al. (53)	15 FE 7 NR 8 R	29.2 (9.4)	4 (^a^)	HD 2 raters IRR 0.85	Acute	Benperidol	All t0: 48.1 (6.6) t1: 35.7 (7.7)	SARS Unchanged	all t0: 16.2 (7.5) t1: 10.0 (4.7)
Germany				BPRS DSM-III-R			R t0: 49.4 (7.8) t1: 32.5 (7.2)		R t0: 15.5 (7.2) t1: 9.3 (4.6)
							NR t0: 46.7 (5.2) t1: 39.3 (7.4)		NR t0: 17.0 (8.5) t1: 10.9 (5.0)

Sevincok and Topaloglu (54)	10 R 7 m, 3 f	24.5 (^a^)	8	NES PANSS DSM-IV	After 2 weeks of washout	Olanzapine	t0: 78.8 ± 19.9 t1: 58.0 ± 13.1	AIMS^a^ SARS^a^	t0: 19.1 (13.2) t1: 14.7 (12.5)
Turkey							*p* < 0.05		n.s.

Smith et al. (55)	37 CH 25 m, 12 f	42.8 (7.6)	88.7 (74.3)	NES slightly modified 1 or 2 or more raters ICC 0.82–0.99	Hospitalized for >1 year	Typicals	t0: 41.9 ± 9.6 t1:^a^	^a^	t0: 27.1 (11.2) t1:^a^; n.s.
						22 pats changed to atypicals			62–67% stable scores no effect of medication
USA				BPRS DSM-III-R					22/37 pats changed to atypicals t0: 35.35 (9.0) t1: 32.55 (11.52)

Tucker and Silberfarb (1)	18 CH or R	33.1 (2.0)	156 (^a^)	4 tests, 1 rater:	On hospital admission	^a^	^a^	^a^	Impairment
				finger agnosia, finger-tip number writing, form recognition, tactile performance					t1: 61.11% pats t2: 44.44% pats
USA				NHSI and SFRSS					“Marked improvement over time”

Torrey (56)	30 CH and R	(In their mid 30ies)	52 (4–82)	2 tests, 1 rater	^a^	Predominantly fluphenazine	^a^		6 pts improved 4 pts worsened
USA				Graphethesia, face-hand test					20 pts stable

Wahlheim et al. (57)	75 R 51 m, 24 f	27.3 (^a^)	104 (^a^)	From NES: motor sequencing and coordination 1 rater	Mostly remitted	^a^	^a^	AIMS SARS	t1: 6.59 (5.36) t2: 5.49 (5.50)
Germany				PANSS SANS DSM-III-R					*p* < 0.05

Whitty et al. (30, 46)	73 FE 103 FE: 66 SCZ 37 others	23.4 (^a^)28.3 (11.4)	26 (^a^) 208 (^a^)	NES and CNE 1. rater CNE 2. raters ICC 0.82	Max. 30 days of medication	Clinical needs	t0: 83.8 (20.1) t1: 58.4 (15.1) *p* <.001 t2 *	^a^ NSS Improvement after 4 years in SCZ pts *p*<0.001	NES *p*<.01 t0: 15.6 (9.7) t1: 12.5 (7.3) CNE n.s. t0: 8.6 (6.5) t1: 7.3 (4.2)
Ireland	64 m, 39 f 89 lateralized, 14 mixed-handed			PANSS DSM-IV			CNI corr NSS *p*<0.0001		

*Authors who published more than one paper on the same group of patients, i.e., at different catamnestic points, are summarized*.

*^a^Information not provided*.

*Numbers in parenthesis: SD, if one number; range, if two numbers*.

*AIMS, Abnormal Involuntary Movement Scale; Barnes, Barnes Rating Scale for Drug-Induced Akathisia; BMS, Brief Motor Scale (59); BPRS, Brief Psychiatric Rating Scale; CH, Chronic schizophrenia patients; CNE, Condensed Neurological Examination (60); CNI, Cambridge Neurological Inventory (61); ESRS, Extrapyramidal Symptom Rating Scale; f, female; FE, first episode; HC, Healthy Controls; HD, Heidelberg NSS scale (29); ICC, intra class correlation; IRR, interrater reliability; m, male; MATRICS, Measurement and Treatment Research to Improve Cognition in Schizophrenia; MCCB, MATRICS Cognitive Consensus Battery; NES, Neurological Evaluation Scale (62); NHSI, New Haven Schizophrenia Index; NR, non-remitters; OC, obstetric complications; PANSS, Positive and Negative Syndrome Scale; Pt(s), patient(s); R, remitters; SANS, Scale for the assessment of negative symptoms; SAPS, Scale for the assessment of positive symptoms; SARS, Simpson Angus Rating Scale; SCZ, Schizophrenia; SFRSS, Schneider First Rank Symptom Scale (63); SKT, “Syndrom Kurztest” (short test for the assessment of cognitive functioning); TD-scale, tardive dyskinesia scale; WCST, Wisconsin Card Sorting Test; NSS: Neurological Soft Signs*.

The number of patients included ranged from 10 to 126, with less than 20 patients in 7 studies; 15 studies dealt with FE patients. Patients suffering from bipolar disorder, major depressive disorder, affective disorders, delusional disorder, substance-induced psychotic disorders, and personality disorders accounted for 21 and 36% of participants in two studies (44, 58); all remaining exclusively consisted of subjects suffering from schizophrenia.

Instruments for the assessment of NSS were mostly standardized and widely used scales, namely the Neurological Evaluation Scale [NES (62)], the Cambridge Neurological Inventory [CNI (61)], the Condensed Neurological Examination [CNE (60)], the Heidelberg Scale [HD (29)], and the Brief Motor Scale [BMI (59)]. The following six exceptions were made: Emsley et al. (41, 42) shortened the NES to 13 items, Madsen et al. (44) employed a standard neurological examination complemented by tests of sensory function and complex motor acts, Mittal et al. (46) used the Quitkin scale (2), Tucker and Silberfarb (1) applied 4, Torrey (56) and Wahlheim et al. (57) two single tests respectively.

Apart from adjustments and exceptions, the different NSS scales and scores are not comparable directly since the number of items differs between instruments as does the scoring (0–1 to 0–3 points) of the individual items. However, items and/or subscales are contrastable, especially with respect to motor sequencing, motor coordination, and sensory integration, which are the most relevant items concerning pathology in schizophrenia. These important components of NSS were assessed in almost all studies integrated in this review.

Ratings were performed by 2–3 raters in half of the studies, achieving overall good interrater reliabilities (see Table 1). One single rater applied the tests in the remaining 50%, which was mainly the case in older studies.

Catamnestic periods extended from a short-term of 2–8 weeks to a medium duration of 6 months to 2 years to long-term follow-ups of 5 and 6 years (see Table 1).

### NSS during Follow-up

Researchers who included HC in their assessments unanimously reported that patients scored significantly worse than healthy comparison subjects [review by Dazzan and Murray (6)]. Table 1 indicates that 35% of all studies in this review compared to HC patients, all of which showed significant differences in NSS scores throughout.

For reasons of better comparison, we grouped all identified studies according to patients’ illness status and their NSS course (Table 2). Independent of the number of patients included and the instruments used for the assessment of NSS, all studies on chronic schizophrenia showed that NSS remained stable or deteriorated, whereas studies on patients with a remitting course found that NSS decreased over time. The pattern also appears in the studies by Schröder et al. (29, 52, 53), in which both chronic and remitting patients participated. There are two exceptions, namely the studies by Jahn et al. (43) and Torrey (56) where mixed samples were included and the respective NSS results on follow-up are heterogeneous. Presumably the divide follows the above model, which, however, cannot be proven or rebuted.

**Table 2:** Follow-up studies in schizophrenia and NSS course (decreasing – stable – increasing).

	Decreasing neurological soft signs (NSS)	Stable NSS	Increasing NSS
First episode	Bachmann et al. (31)	Behere (34)	
		Boks et al. (35)	Boks et al.: with increasing medication (35)
	Chan et al. (37): pats without negative symptoms	Chan et al. (37): pats with negative symptoms	
	Chen et al. (39): motor signs only	Chen et al. (39): all except motor signs	
	Cuesta et al. (40): clinically improved pats (16%)	Cuesta et al. (40): stable pats (84%)	
	Emsley et al. (41): motor sequencing initially	Emsley et al. (41)	
	Emsley et al. (42)		
	Mangot and Sawant (45)		
		Madsen et al. (44)	Madsen et al. (44): with deteriorating course
	Ojagbemi et al. (47): in good responders	Ojagbemi et al. (47): only motor sequencing; in poor responders	
		Scheffer (51)	
	Prikryl et al. (48–50)		Prikryl et al. (48–50): with negative symptoms after initial decrease
	Whitty et al. (30, 58)		

Remitting course	Schröder et al. (29)		
	Schröder et al. (52)		
	Schröder et al. (53)
	Sevincok and Topaloglu (54)		
	Tucker and Silberfarb (1)		
	Wahlheim et al. (57)		

Chronic course		Buchanan et al. (36)	Chen et al. (38)
		Schröder et al. (29)	Mittal et al. (46)
		Schröder et al. (52)	
		Schröder et al. (53)	

Mixed group	Jahn et al. (43)	Jahn et al. (43)	
		Smith et al. (55)	
	Torrey (56)	Torrey (56)	Torrey (56)

The picture on FE patients is less clear. Whereas patients’ NSS status improved in several studies (30, 31, 42, 45, 58), the patients’ cohort fell into two subgroups in the majority of studies, mostly with one subgroup exhibiting an improving and another a stable NSS course over time.

Only Boks et al. (35) and Madsen et al. (44) reported a division into two subgroups with stable and deteriorating NSS, and Prikryl et al. (48–50) described two subgroups with improving and worsening course, respectively. No change except for worsening of the glabellar reflex was observed in one study (51).

### NSS Subscales during Follow-up

Change in total NSS—if present—was mostly accounted for by change in the motor system subscales, i.e., motor coordination and motor sequencing (29, 31, 38, 49, 51, 58). Cuesta et al. (40) reported an improvement in all subscales except for frontal signs.

Emsley et al. (41) reported a decrease of motor signs during the first 3 months of treatment with a subsequent return to initial values. Accordingly, in their second study, the group (42) did not note a drop in motor signs despite of a significant decline in total NSS. Other authors divided their samples into remitters and non-remitters and reported a decrease of motor signs being related to a remitting course (50 plus sensory integration; 29 plus spatial orientation). Likewise, in a FE study (31) subgroups arose after 1 year, which were distinguishable by the subscales motor coordination, motor sequencing, and sensory integration. The sensory integration subscale also added to an overall change in NSS scores in two other studies (51, 38), right-left and spatial orientation did so in one (31). Moreover, one group detected a non-significant decrease of the above-indicated subscale scores (54) and another group described a concordant finding for sensory integration (34).

Among the groups that did not detect any NSS change over time, Smith et al. (55) pointed out the relatively high stability of motor signs as opposed to the low stability of sensory integration signs in chronic patients. In FE individuals two groups found stability of motor signs (39, 47).

Chen et al. (38) reported deterioration of motor coordination, sensory integration, and disinhibition in a complete sample of chronic patients. Unfortunately, Boks et al. (35), Mittal et al. (46), and Whitty et al. (30) did not give any information on subscales when there was an overall NSS deterioration. In the study by Madsen et al. (44), NSS increase was mostly related to corticospinal tract signs.

### NSS, Symptoms, and Other Clinical Variables

Medication influences both clinical variables and NSS (see below). Nevertheless, the relationship between NSS and clinical variables will be discussed separately.

Two studies did not assess or indicate psychopathology. The Scale for the Assessment of Negative Symptoms, and the Scale for the Assessment of Positive Symptoms were used by two groups, completed by the Present State Examination (PSE-9) once, whereas all remaining researchers employed the Positive and Negative Syndrome Scale (PANSS) or the Brief Psychiatric Rating Scale (BPRS). The respective scores at study inception and follow-up are given by 13 authors, whereas, 6 reported incomplete data (see Table 1). Scores are essentially comparable in the sense that (a) scores assessed with the same instrument fall into the same range, (b) the absolute follow-up scores are even similar, (c) acutely ill patients’ score decrease over time, and (d) scores of chronic patients or of those who were assessed in a stable condition remain widely unchanged.

Several authors have reported an improvement of NSS in parallel to a *decrease of symptoms or treatment response*, respectively (1, 29–31, 40, 42–44, 46–52, 54, 56–58). This pattern was even found in patients without symptom change (44) and in a subgroup without negative symptoms (34). Furthermore, associations of remission and motor signs were described (44, 58, 30), whereas remitters in the studies by Prikryl et al. (48–50) exhibited lower total, sensory integration, and motor sequencing scores. Also severity of illness and lower social functioning (44) or severity of illness, except for motor sequencing, correlated with NSS (47). Other researchers did not find an association between NSS with symptoms in first-episode (35, 45) or chronic patients (36, 55). In two studies, even a deterioration of NSS in chronically ill (38) and of corticospinal functions in non-remitting FE patients was detected (44).

*Positive symptoms* were only addressed separately in a few studies. Scheffer (51) reported significant associations of NSS change in motor coordination, sequencing of complex motor acts, and sensory integration with change in the positive subscale (and total BPRS score, as discussed above). This is supported by findings of significant positive correlations between NSS and positive symptoms in two studies (45, 46) and of balance and positive symptoms in another study (41). Results by Chen et al. (38) did not underline the aforementioned findings. Even after a split into high and low positive symptoms no association with NSS emerged.

Researchers reported more extensively on *negative symptoms* compared to other symptoms present at the catamnestic examination. Many groups found a positive or increasing correlation over time between total NSS scores and negative symptoms (30, 37, 40, 42, 44, 48–53, 58), as well as correlations between motor signs and negative symptoms (39, 47). One group described partial correlations of negative symptoms with primitive reflexes (34). Yet others reported an association of NSS with discomfort (46) or with negative and disorganized symptoms (47). Some groups depicted constancy of negative symptoms and NSS over time (34, 54, 55) in diverse patient samples and even in individuals with variations of NSS. Whitty et al. (30) even posit that negative symptoms rather than positive or general psychopathological symptoms may predict NSS.

Some authors considered *further symptoms* in their reports. They found an association between rapid movement or convergence and depression or anxiety (41), of total NSS with disorganization symptoms (42) on the one hand and a negative correlation of NSS and discomfort (46) on the other hand.

The meta-analysis by Chan et al. (16) already reported a strong relationship between NSS and symptoms in general. This notion is confirmed and extended by longitudinal studies which argue that the association even increases over time, the longer the more, and holds true mostly for negative symptoms.

Results on outcome are thus divergent: authors report (a) either no difference between good and poor outcome patients with respect to motor signs (39) or (b) better outcome measures in patients who complied with treatment and exhibited decreasing NSS scores (31); along these lines, remission was inversely correlated to NSS as described by two groups (29, 48, 49). In terms of prediction, bidirectional relationships are possible: NSS predict symptomatology (46), positive and negative symptoms, as well as other parameters predict increasing NSS (30). From the perspective of outcome, there seems to be a close relationship to NSS and psychopathology, in the sense that both higher NSS and higher psychopathology are related to a worse outcome.

*Cognitive functioning* was addressed in several studies. Chan et al. (37) did not find any group*time effect with NSS and overall cognitive parameters. However, over time, NSS correlated with poorer performance on the letter–number span test. Their group also reported paralleling trends of NSS and Winsconsin Card Sorting Test (WCST) perseverative errors in both patient groups as well as higher scores in logical memory, delayed logical memory, and WCST category, which authors relate to worse motor coordination and total NSS scores. In another study (42), working memory amelioration was predicted by sensory integration and motor coordination, whereas motor sequencing was predicted by working memory. Jahn et al. (43) even found correlations between NSS and all subscales of the modified WCST. Another study from Germany (52) reported a parallelizing improvement of both NSS and cognitive parameters, namely d2, Tower of Toronto test, SKT: delayed recall (Syndrom-Kurzztest, short assessment of cognitive performance), and SKT: delayed recognition. Further nonsignificant improvements occurred in the Tower of London test and SKT: immediate recall. Along these lines worsening of higher cognitive functioning paralleled higher hard signs (30, 46), and patients with NSS—as opposed to those without—exhibited negative symptoms and cognitive disorganization (57). Respective correlations emerged in cross-sectional research (64–68).

Patients included in the reviewed study were of relatively young *age*. None of the authors discussed age with respect to NSS. The same holds true for *gender*. The literature does not report any influence of sex on NSS. Neither did we detect any influence. *Family history* was addressed by Madsen et al. (44) who found an NSS deterioration in a subgroup with first-degree relatives who suffered from a psychiatric disease. The same authors reported a worsening of NSS in patients with a history of *obstetric complications*. A relationship between *level of education* and NSS on follow-up was reported by two groups (39, 58, 30).

*Handedness* was examined where indicated in Table 1. Whereas our group did not detect any influence of handedness on NSS, Whitty et al. (58, 30) found higher NSS in mixed handers. The latter may be related to a common basis of both, mixed-handedness and NSS, consisting of still ill-defined neurological abnormalities.

Several authors addressed the *duration of untreated psychosis* (DUP) which was positively associated with NSS (39, 56, 58, 30) and negative symptoms or with motor symptoms only (39, 41). Similar associations arose with the overall *duration of illness* (45, 46, 55). Whitty et al. (58, 30) even posit that the DUP predicts catamnestic levels of NSS.

*Alcohol and substance use* were exclusion criteria in almost all studies, thus making the study population less representative. When this was not the case, alcohol did not influence NSS (57) or, together with negative symptoms, predicted higher NSS scores (58, 30). The latter finding is supported by the literature (69), stating that drug and alcohol abuse are associated with more neurological abnormalities in schizophrenia.

### NSS, Medication, and Side-Effects (SE)

Patients were treated with typical neuroleptics in 10 studies, with atypical compounds in 5 studies, and with any of both or a mixture in the remaining 11 studies, where authors not always gave the respective information or, e.g., just displayed chlorpromazine-equivalents.

Five groups reported on a sample of antipsychotic-naïve or about 90% medication-naïve patients; another two groups included antipsychotic-naïve individuals but to a lesser extent (51, 29). A washout phase prior to starting the study was reported by two researchers (46, 54).

Dosages, where given, are not comparable between studies due to the different ways of reporting. Cuesta et al. (40), who used different antipsychotics or combinations, explicitly stated that there was significant NSS improvement over time independent of the type of medication, whereas Smith et al. (55) observed a trend toward lower NSS scores on atypical antipsychotics.

Whether or not antipsychotic medication exerts an effect on NSS can neither be proven nor disproven because the effect can only be generated indirectly. Although there is no indication regarding different effects of typical versus atypical compounds, evidence from the reviewed studies points toward different medication effects:

(a) a positive influence of medication on NSS in terms of a decrease mostly stems from FE patients (1, 31, 40, 45, 51, 58), but also from those with a longer-standing illness (46, 54, 55). Others found that medication intake was associated with less motor signs as opposed to a non-medicated status (30, 35, 58).
(b) partial positive effects were reported as well in FE and remitting versus non-remitting patients (29, 37, 47, 52, 53, 56, 57).
(c) no relationship arose between NSS daily dose or type of antipsychotic compound (44, 44, 49, 55).
(d) medication non-response and non-remitting NSS scores were related in studies on FE (31, 34, 35, 44, 48, 49) and on chronic patients (36). One group reported that the association pertained to all subscales except sensory integration.
(e) two studies reported a relation between lifetime exposure to medication and less NSS change or even NSS increase in medication non-responders (35, 44).

In summary, medicated patients seem to fall into at least two subgroups, namely one which improves clinically and in terms of NSS, and another which exhibits stable and/or worsening psychopathology as well as NSS. Possibly, the effect of medication may be related to the stage of illness and the overall magnitude of NSS which have been said to be predictors toward response to medication (70). Despite the diverse results, there is thus room for supporting the notion that antipsychotic compounds exert a protective effect on NSS (6). Already, Heinrichs and Buchanan (14) argued similarly by reporting that medicated patients exhibited fewer signs.

Consequentially, the relationship between clinical response to medication and NSS improvement—or non-improvement, respectively—leads to the assumption of a common underlying factor. For example, this may be the effect of antipsychotics on brain structure as reviewed by Scherk and Falkai (71). Ultimately though, the link between medication, brain structure, and NSS has not been located yet.

Side-effects of medication were assessed by 15 groups, using widely accepted instruments. Six authors indicated that no SE were present, five others did not give any results, hereby suggesting that non-reporting is due to a lack of significant pathology. A minority of six groups reported associations between NSS and extrapyramidal side-effects: a positive correlation on follow-up (47), a relationship to motor signs (44), to motor signs on 2-year follow-up (41), to sensory integration (45), or to stable, i.e., higher NSS (57). A significant increase of tardive dyskinesia on follow-up was observed in haloperidole treatment (36).

### NSS and Ethnicity

The presented follow-up studies on NSS in schizophrenia were performed in different countries on four continents, namely, (North) America, Europe, Asia, and Africa. So far, study results do not show any pattern in favoring or contradicting an association of NSS and ethnicity, although more studies are warranted. For now, there is reason to assume that NSS may be remarkably similar across countries and ethnicities (72).

### Methodology of Studies

Representativeness of the reviewed studies is high for the following reasons: only two research groups included diagnostic groups other than schizophrenias, the majority of researchers followed patients for at least 6 months, 47% of the studies focused on FE patients, and interrater reliability or intraclass correlation was high overall. Different assessment instruments do not seem to exert an influence: although different researchers have used different instruments, these were standardized and featured the same dimensions of NSS. Moreover, there is agreement in the literature on the relative importance of the different groups of NSS: motor signs, i.e., motor sequencing, complex motor tasks, and motor integration are of overriding importance as they usually account for the largest part of the total NSS score. Sensory integration on the other hand plays a minor role.

### The Trait versus State Debate

As schizophrenia has a genetic basis in up to 50% of cases (9), NSS have been looked at as endophenotypes, i.e., markers which help to discover the underlying genetic basis. There is an assumption that the amount of NSS and the abnormality of the single sign may even discriminate sporadic and familial schizophrenia with more pronounced abnormalities in familial cases (51). Along these genetic lines, many studies have reported more NSS and higher item scores in patients’ relatives compared to HC; the closer the relative, the more explicit the abnormalities (21). On the other hand, according to Kinney et al. (73) and Rossi et al. (60) NSS have a small to modest share in the liability toward schizophrenia. Gureje Gureje (74) even stated that NSS might not be specific to schizophrenia but related to obstetrical complications in general. Madsen et al. (44) may reconcile the opposite views: in their study differences between patients and healthy subjects regarding frontal, cerebrospinal, and temporo-parietal functions became larger over time—which was more prominent in patients with a positive family history. Similarly, Hyde et al. (75) claim that “frontal release signs” are influenced by both genetic and environmental factors.

Meehl (76) stated that NSS can only be considered to represent genetic markers if:

1. they are specific for schizophrenia, i.e., discriminate patients from HC and patients with other psychiatric diseases;
2. siblings show at least a tendency for differing from healthy subjects;
3. acutely ill and remitted patients are comparable; and
4. schizophrenia patients with varying degrees of severity do not differ regarding NSS.

Meehl’s first requirement for a genetic marker partially holds true in NSS. There was congruence among the reviewed studies that patients exhibit more NSS than HC. This is supported by the literature including FE patients (6, 77). However, NSS can also be detected in patients suffering from a number of other psychiatric diseases including bipolar disorder, obsessive–compulsive disorder, borderline personality disorder (78–80).

Meehl’s second postulation is fulfilled. NSS were also detected in siblings, but were qualitatively and quantitatively less distinct, i.e., taking an intermediate position between HC and patients. This represents a strong argument for a trait or hereditary component as backed up by the literature on patients’ first-degree relatives (20), on twins (21–24), and on off-spring (25–27).

Requirement three demands comparability between acutely ill and remitted patients. Some studies in our review argue in favor of comparability by reporting completely stable NSS over time. This was the case even in four FE studies (35, 39, 41, 51) and in studies on chronic patients (36, 39, 55). The other studies reported differences between subgroups. However, the above-indicated FE patients presented with a long duration of untreated illness and might already have crossed the border toward chronicity, e.g., in the study by Chen et al. (39) where DUP amounted to 474 ± 768 days. Along these lines, some authors suggested an interrelationship between NSS and duration of illness (81, 83, 84). Moreover, there was one report (38) on a clear deterioration of NSS in chronic patients and a further one on patients worsening on a trend level (44), related to a non-remitting course, a family history of psychotic disorder in a first-degree relative, obstetric complications, and male gender.

Further arguments in favor of non-comparability between acutely ill, remitted, and chronically ill patients arise from studies which showed NSS improvement over time in most or all patients, differences in phases of the disease and other varying factors notwithstanding. Several follow-up studies found NSS improvement, especially in FE patients (30, 31, 42, 45, 49, 58) with respect to subscales in FE (16, 34), in patients with a remitting course (1, 29, 44, 49, 56, 57) with significant effects in two of the cited studies, and in patients who were unmedicated prior to study inclusion (46, 54). Moreover, in the study by Sevincok and Topaloglu (54), patients who were unmedicated at first assessment and medicated at the second displayed an absolute decrease of NSS—possibly due to the small number of patients the results did not reach statistical significance. In all probability, compliance plays a role toward improvement (31). As adherence most likely is particularly high in patients who participate in a study, compliance presumably was present in the presented studies. Thus, at least at the onset of the illness and in remitting subtypes an NSS state component is present and expressed by fluctuations of NSS scores. Given the number of studies reporting fluctuating and decreasing NSS in FE and remitting patients, and the fact that only chronic patients experience stability or deterioration of NSS, the state component cannot be ignored. The fact that patients’ samples mostly divide into those with stable NSS and improving NSS at an early stage during their course of illness, is in line with schizophrenia not just representing one homogeneous disease but a group of entities, namely the “group of schizophrenias” as Bleuler put it (82). As the existence of subgroups is not debatable (83, 85)—subtypes may represent another argument for the existence of an NSS state feature in some patients but not others. Unfortunately, subtypes of the disorder and their influence over time were discussed by Schröder et al. (29) only.

The fourth claim by Meehl states that schizophrenia patients with varying degrees of severity should not differ regarding NSS. Severity of patients’ illness in the different studies can either be compared by authors’ classification as FE, remitting, and chronic course or by looking at psychopathology. Whenever scores of well-known scales such as BPRS or PANSS were disclosed, their range was comparable. The latter comparison seems more valid because the former already is based on symptoms. With respect to psychopathology and NSS, differing patterns arise in those studies, which gave all necessary information. Some authors report a paralleling course of NSS and psychopathology in all patients (30, 31, 40, 42, 46, 54, 58) or in subgroups (44, 48–50, 52). Others describe decreasing symptoms (except negative symptoms) but stable NSS (34) or the opposite, namely stable symptoms and decreasing (34, 53) respectively increasing NSS (38). Yet, further studies found unchanged symptoms as well as NSS over time (35, 36).

With this diffuse pattern, Meehl’s fourth demand is clearly not fulfilled. However, the pattern speaks in favor of a state component in NSS and the necessity to consider the stage of illness, as Smith et al. (55) reasoned: if a state dimension was predominant, the timing of NSS assessment would be important. This argument also calls into question the predictive value of NSS which was proposed by earlier authors (66, 87).

Thus, only two of four of Meehl’s claims are fulfilled and NSS qualify for both, a stable, and early-acquired trait and a fluctuating state component. The latter may possibly be related to the stage of the disease as well as to treatment.

## The Nature of NSS

In accordance with the literature, we argue that the NSS dimensions might be related to a neurointegrative defect in general (86), to dopamine-dependent pathways (87), or even more specifically to certain brain regions and circuits connecting them (11, 14, 88), be it at a subcortical level (9, 89) or in terms of a cortical-subcortical pathway (90). Among the circuits in question, the fronto–striatal–thalamic route emerges as the most relevant toward motor signs which again seem to contribute overridingly toward overall NSS scores. According to our understanding the fronto–striatal–thalamic pathway is being complemented by integration of the cerebellum (91–93), thus forming the cortico–cerebellar–thalamo–cortical circuitry suggested by Andreasen et al. (94). Interestingly, Kong and colleagues (95) detected changes of the basal ganglia in ultra-high-risk subjects prior to manifestation of the disease. Changes of the basal ganglia in relation to NSS were already reported in 1998 (53) by applying SPECT. The authors showed that upregulation of dopamine d2 receptors under neuroleptic treatment may be involved.

These findings may partly explain the above cited effect of antipsychotics on brain structure as reviewed by Scherk and Falkai (71): typical and atypical antipsychotics exert varying effects on cortical gray matter and on almost all subcortical structures. The thalamus increases with both types of neuroleptics. This may reflect protective or regenerative processes related to the stabilization of NSS, especially of motor signs which most likely pertain to the cortico–thalamo–cerebellar–cortical circuit.

As far as the remaining NSS are concerned, Carter et al. (96) suggested a disturbed integration of the sensory systems. Dazzan et al. (6, 88) concluded that agreement concerning the neuro-dysfunctional basis of NSS is still missing.

However, similarities between longitudinal studies, in terms of general NSS elevation and change of NSS scores over time, clearly support a combination of state and trait aspects or a Janus-faced nature of NSS in schizophrenia. Unfortunately, the question of what underlies the state component has to remain open. At this point, innate and adaptive immune response may come in to play. As far as innate immune system alterations are concerned, prenatal maternal infections (97, 98) as well as obstetric complications, neonatal hypoxia, and brain injury lead to recruitment of cytokines which mediate inflammatory processes or represent a proinflammatory immune state in itself. Also, early strikes hitting the immune system may lead to a lifelong change in immune response and low-level neuroinflammation. For example, cytokines activate indoleamine 2,3-dioxygenase, which influences tryptophan, kynurenin, and serotonin in the central nervous system. As a consequence, serotonergic, noradrenergic, and glutamatergic neurotransmission may be modified (99). Changes in other inflammatory biomarkers (macrophages/monocytes, reduction in T cell numbers and proliferation, and alternations in T-helper cell 1 balance) may activate microglia, i.e., the intracerebral macrophages (98). Alterations of the immune response *via* inflammatory processes may also be related to infectious agents, which can cause acute or latent infections, namely, viruses and parasites, such as Human Herpesvirus 2, Borna Virus, *Chlamydophila pneumoniae, Chlamydophila psittaci*, and *Toxoplasma gondii* (100). Latent infections and reactivation of latent infections seem to be associated with acute disease phases in schizophrenia (31, 101, 102). Moreover, Human Endogenous Retrovirus may be activated in the genome by microbiological agents, immune mediators as mentioned above, and multiple other activators of the cell and thus be linked to schizophrenia in some individuals (103–106).

All of the above-indicated factors may cause dysregulation of dopamine and further neurotransmitters, either directly or indirectly, and lead to impairments in cortico-subcortical networks.

These findings on infection and inflammation very much support a two-hit model of schizophrenia with a genetic or early acquired as well as a second-adapted factor forming the base of NSS and their trait and state aspects.

Above and beyond open questions on the nature of NSS, an integrative view of the different motor symptoms is warranted (107), as well as a taxonomy of symptoms in schizophrenia encompassing the sensory–motor dimensions.

## Conclusion

The literature on NSS in schizophrenia presents at least partially diverging results. On the one hand, research speaks in favor of NSS as being a stable entity, i.e., a trait: NSS are more pronounced in patients compared to their relatives; they are present at disease onset; there is a relationship with the more stable symptoms, namely negative and cognitive ones. Duration and chronicity of illness have also been linked with stability of NSS. On the other hand, there are arguments supporting a fluctuating aspect, especially of motor signs. Follow-up studies—as summarized in this paper—have reported modulations of NSS scores over time, especially in correlation with clinical improvement, in FE patients, and in the relapsing–remitting subtype. Taken together, the evidence on NSS reconciles the dispute by suggesting that both components are involved, e.g., a trait or baseline component as well as a state component associated with psychotic exacerbations.

This underlying state-trait dichotomy of NSS is hardly influenced by the type of medication, supposedly apart from the lifetime load. Furthermore, in a state-trait feature, only the presence of the trait can be used as an endophenotype or a predictor, but not the quantitative expression, i.e., the state. The latter however, is called into question by studies which detected a paralleling course of symptoms and NSS, meaning that the volatility in terms of NSS decrease may serve as an outcome predictor.

Future research should raise the question of whether or not testing of NSS can be restricted to motor and sensory signs because these are the major subscales which account mostly for fluctuations in NSS scores. Moreover, apart from identifying the circuits which underlie the different NSS, researchers should study the mechanisms involved in the state component, i.e., undulations of NSS scores.

## Author Contributions

SB wrote and edited the manuscript. JS critically revised the different versions of the manuscript.

## Conflict of Interest Statement

The authors declare that the research was conducted in the absence of any commercial or financial relationships that could be construed as a potential conflict of interest.
